# Step techniques for backward and sideward sprint starts used by high-level male soccer players

**DOI:** 10.1016/j.heliyon.2021.e07333

**Published:** 2021-06-18

**Authors:** Takahiko Sato, Yusuke Fukuhara, Tadao Isaka

**Affiliations:** a Faculty of Rehabilitation, Biwako Professional University of Rehabilitation, Japan; b Graduate School of Sport and Health Science, Ritsumeikan University, Japan; c Faculty of Sport and Health Science, Ritsumeikan University, Japan

**Keywords:** Sprinting, Multi-directional movement, Soccer

## Abstract

From standing in a parallel stance, two common techniques for sprint starts are forward and false steps. In the forward step technique, athletes take a first step in the sprinting direction; in the false step technique, the first step is in the opposite direction to the sprinting direction. Although the false step technique, including a redundant step, has generally been considered as an inferior technique, athletes habitually use it to start sprinting in a forward direction. The present study aimed to clarify which step technique is habitually used by high-level male soccer players when they start sprinting in a backward or a sideward direction. From a stationary standing position, 15 male soccer players were instructed to sprint backward and rightward three times each, and the step techniques used to start sprinting were recorded. In the backward sprint start trials, 2 trials were done using the forward step technique and 43 using the false step technique. In the rightward sprint start trials, 27 trials were done using the forward step technique and 18 using the false step technique. While the false step technique was used significantly more than the forward step technique in the backward sprint start trials (p < 0.001), no significant difference was found between the use of either technique in the rightward sprint start trials (p = 0.18). The results demonstrate that high-level male soccer players habitually use the false step technique in a backward sprint start and use both techniques with similar frequencies in a sideward sprint start.

## 1 Introduction

In field- or court-based sports, such as various kinds of football, athletes repeatedly sprint to get a loose ball or evade opponents. The sprinting distance in these sports averages about 20 m [1, 2, 3], and the time taken to complete a short-distance sprint is shorter in athletes on higher-level teams [4, 5]. For the performance of a short-distance sprint, the ability to rapidly accelerate is more significant than the maximal sprint velocity, because the distance is insufficient to allow an athlete to fully accelerate. Thus, in these sports an improvement in the sprint start technique that can shorten the time required for a short-distance sprint contributes to the athlete's performance and the team's success.

From a standing position with a parallel stance, the two most common techniques for sprint starts in a forward direction are forward and false steps. In the forward step technique, athletes take a first step in the sprinting direction. Conversely, in the false step technique, an initial step, which is termed a “false step,” is taken backward before the first step in the sprinting direction. The false step technique has been generally considered inferior to the forward step technique because it includes an unnecessary step in a direction opposite to the sprint. However, most previous studies comparing the performance parameters of short-distance sprints (up to 5 m), such as sprinting time or velocity, reported that the false step technique outperforms the forward step technique [6, 7, 8, 9, 10, 11].

These previous studies suggested that the false step was superior due to the following two factors. The first factor is the time required to position the center of mass of the whole body (COM) anterior to the center of pressure of ground reaction forces (COP). In the forward step technique, this is achieved by tilting the body forward to adjust the COM; in the false step technique, it is achieved by stepping back to adjust the COP. Compared to the forward displacement of COM in the forward step technique, the shorter time required to step back in the false step technique is considered to be more beneficial for a short-distance sprint. The second factor considered is an enhancement of the ground reaction force (GRF) acting on the foot that has stepped back when it pushes off, which is caused by the stretch-shortening cycle and kinematic energy of the stepping foot. However, previous studies focusing on the sprint start from a staggered (or split) stance in which the feet are positioned one behind the other; the short-distance sprint time using the false step technique was not shorter than that using the forward step technique [11, 12]. These studies suggested that it is the quick move of COP, rather than the GRF enhancement, that mainly provides the superiority of the false step technique.

Athletes in field- or court-based sports start sprinting in various directions on the basis of relevant visual cues, such as the ball's movement or an opponent's unexpected moves. However, almost all previous studies comparing sprint performance between the forward and false step techniques focused only on sprint starts in a forward direction [6, 7, 8, 9, 10, 11, 12, 13, 14]. To our knowledge, our preliminary report is the only study comparing sprint performance between the forward and false step techniques in a direction other than forward [15]. Hence, further research is warranted on sprint start techniques in a sideward or backward direction. To start sprinting in a backward or sideward direction, the athlete must turn their body toward the sprinting direction. Therefore, which step technique is superior for a sprint start in a sideward or backward direction may differ from that in the forward direction. The stream of research focusing on the superiority of these step techniques in forward sprint starts was begun with a report by Kraan et al. [14], and their research had been conducted because they found it noteworthy that the sprint start is always accompanied by a step backward. To begin investigating the superiority of these step techniques in backward and sideward sprint starts, the present study aimed to clarify what step techniques athletes habitually choose when they start sprinting in a backward or sideward direction. The choice of step technique can vary among sexes, sport types, and athletic levels. In the present study, we focused on high-level male soccer players who had likely gained enough experience and exposure to have fixed sprint start preferences.

## 2 Materials and methods

### 2.1 Participants

Fifteen male soccer players from a top-level college soccer team, who practice six times a week, volunteered to participate in the present study. Right fullback players were recruited for the present study because they are likely to perform the backward and sideward sprint starts with higher frequency compared with other positions. The mean value and standard deviation of ages, heights, and body masses of the participants were 21.1 ± 1.5 years, 1.73 ± 0.04 m, and 66.7 ± 4.1 kg, respectively. The dominant foot of all participants was the right foot. The Human Ethics Committee of Ritsumeikan University approved all procedures in the present study (ID: BKC-IRB-2011-019), and informed written consent was obtained from each participant before the experiments were carried out.

### 2.2 Experiments

**Figure 1:**
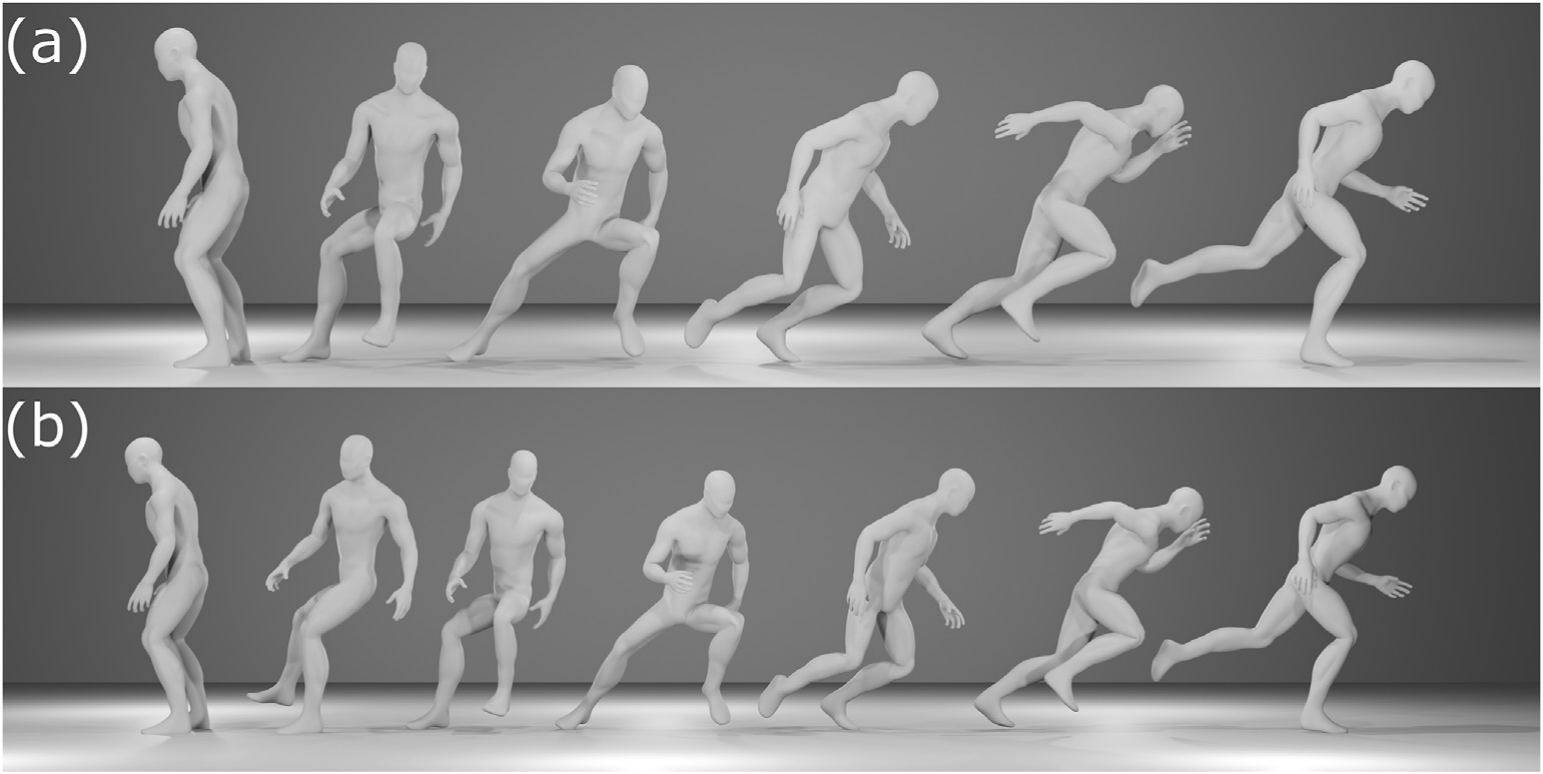
Side view of the backward sprint start using (a) the forward step technique and (b) the false step technique.

**Figure 2:**
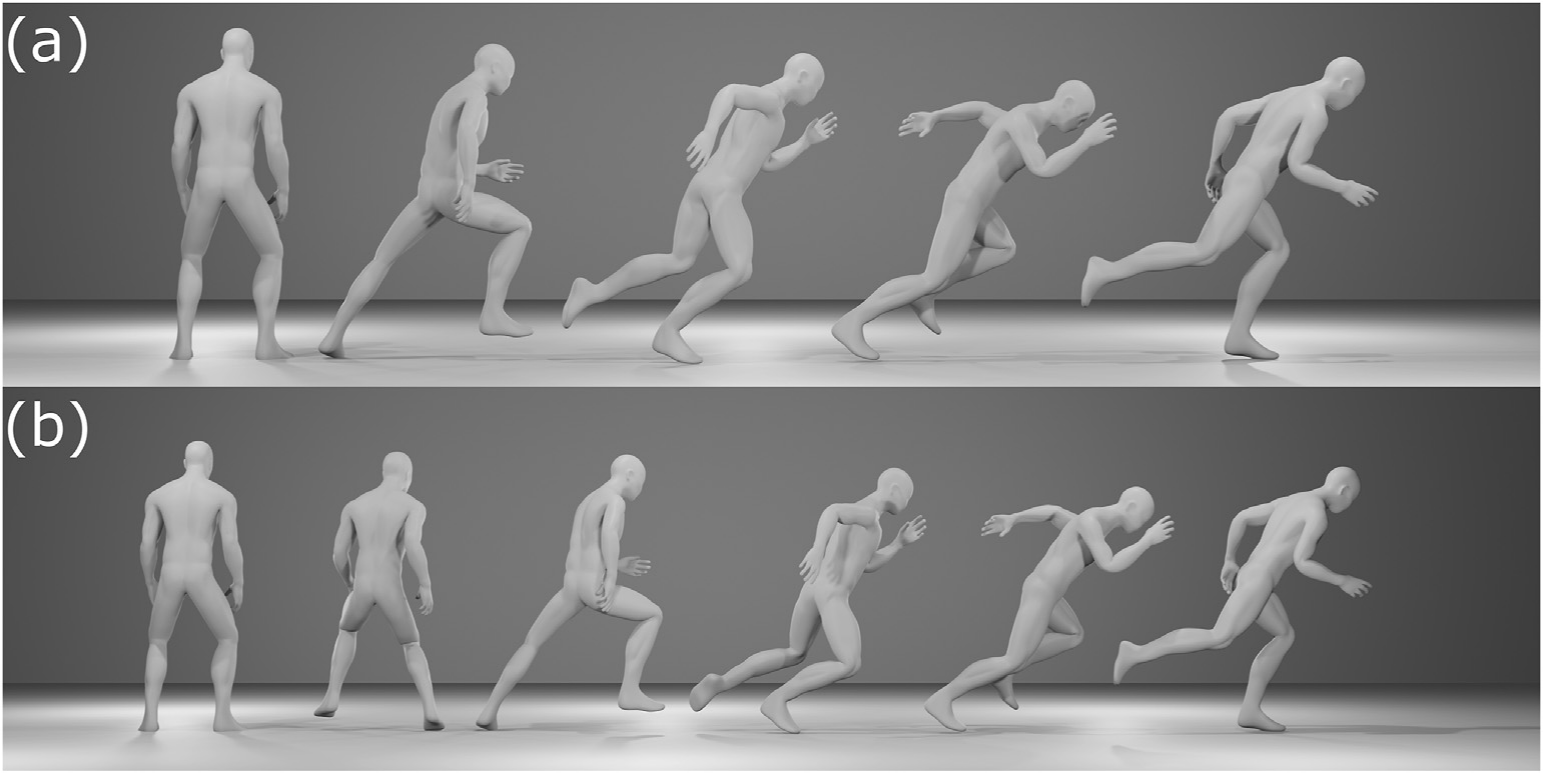
Back view of the rightward sprint start using (a) the forward step technique and (b) the false step technique.

Following an individualized warm-up, each participant performed 5-m sprints in a rightward and a backward direction from a stationary standing position. Turning direction in the backward sprint start was a counter-clockwise direction as viewed from above. The sprinting and turning directions were determined based on the movements usually performed when a right fullback defends a dribbler from the attacking side. The starting position and stance width were determined according to each participant's preference in order to quickly start sprinting. The participants self-initiated the sprint after a ready signal. No instruction was provided regarding the steps for the sprint start in either direction. The participants performed three trials of each sprint direction at their maximal effort. The participants did not observe the other participants during testing. The steps used for the sprint starts were recorded by a high-speed video camera sampling at 300 Hz (EXLIM EX-F1, Casio Computer Co., Ltd., Tokyo, Japan). All trials were categorized as using the forward step, false step, or other techniques based on the step sequence used for the sprint start. The step sequences were observed frame by frame on the recorded videos. For the backward sprint starts, a trial in which the participant's left foot was moved toward their back side while turning their whole body was categorized as using the forward step technique; a trial in which their right foot was moved toward their front side as a “false step” before moving their left foot toward their back side was categorized as using the false step technique (Figure 1). For the rightward sprint starts, a trial in which the participant's right foot was moved toward their right side as an initial step was categorized as using the forward step technique; a trial in which their left foot was moved toward their left side as “false step” before moving their right foot toward their right side was categorized as using the false step technique (Figure 2). For both sprinting directions, a trial in which the participant used a step technique other than the forward and false steps was categorized as “other.” To confirm intra- and interobserver reliabilities for identifying the techniques, three observers were asked to categorize the techniques three times using 10 randomly chosen videos. All observers gave the same categorizations three times for all 10 videos, and no conflicts in categorization were noted among the observers. After confirming intra- and interobserver reliabilities, one of the three observers conducted categorization for all trials.

### 2.3 Statistical analysis

Fisher's exact test was utilized to compare the frequency of each step technique between the two sprinting directions. The number of times each technique was used in each springing direction was compared by a chi-squared test of goodness of fit. The statistical significance level was set at 0.05 and adjusted using the Bonferroni correction in the multiple comparison. Statistical analyses were conducted using IBM SPSS Statistics 27 (IBM Corporation, New York, USA).

## 3 Results

In the 45 trials of backward sprint starts performed by the 15 participants, there were 2 trials using a forward step technique and 43 using a false step technique (Table 1). In the rightward sprint start trails, there were 27 trials using a forward step technique and 18 using a false step technique (Table 2). Using Fisher's exact test, a significant difference was found in the frequencies of these step techniques between the backward and rightward sprint starts (p < 0.001). While in the backward sprint start trials there was a significant discrepancy in the use of the forward and false step techniques (p < 0.001), no discrepancy was found in the rightward sprint start trials (p = 0.18).

**Table 1:** Step techniques used for backward sprint starts.

Trial	1^st^	2^nd^	3^rd^
Sub. A	False	False	False
Sub. B	False	False	False
Sub. C	False	False	False
Sub. D	False	**Forward**	False
Sub. E	False	False	False
Sub. F	False	False	False
Sub. G	False	False	False
Sub. H	**Forward**	False	False
Sub. I	False	False	False
Sub. J	False	False	False
Sub. K	False	False	False
Sub. L	False	False	False
Sub. M	False	False	False
Sub. N	False	False	False
Sub. O	False	False	False

**Table 2:** Step techniques used for the rightward sprint starts.

Trial	1^st^	2^nd^	3^rd^
Sub. A	**Forward**	**Forward**	False
Sub. B	False	**Forward**	**Forward**
Sub. C	**Forward**	**Forward**	False
Sub. D	**Forward**	**Forward**	**Forward**
Sub. E	**Forward**	False	False
Sub. F	**Forward**	**Forward**	False
Sub. G	**Forward**	**Forward**	False
Sub. H	False	**Forward**	**Forward**
Sub. I	**Forward**	False	**Forward**
Sub. J	False	False	**Forward**
Sub. K	**Forward**	False	False
Sub. L	False	**Forward**	**Forward**
Sub. M	**Forward**	False	**Forward**
Sub. N	False	False	**Forward**
Sub. O	False	**Forward**	**Forward**

## 4 Discussion

The purpose of the present study was to clarify the step technique habitually used by high-level male soccer players to start sprinting in backward and sideward directions. A statistical analysis revealed that the false step technique was used significantly more than the forward step technique in backward sprint starts. In contrast, there was no difference between the frequency with which the two techniques are used in rightward sprint starts. These results indicate that high-level male soccer players habitually use the false step technique for a backward sprint start, and there is no bias in choosing a technique in a sideward sprint start.

Compared with the forward step technique, the false step technique used in most backward sprint start trials may enable quicker sprint starts in the backward direction based on the knowledge about sprint start in the forward direction. In a previous study focusing on sprint starts in a forward direction, the superiority of the false step technique was caused by the COP-COM distance quickly generated by the “false step” [8, 9, 10, 11, 12, 13]. To gain greater acceleration, the COP must be posterior to the COM, and a large COP-COM distance is preferred. The false step is redundant but enables athletes to gain greater early acceleration compared to the forward step technique [9, 12, 14]. As in the forward sprint start, a backward sprint start may benefit from an advantage in acceleration provided by the false step.

In backward sprint starts, athletes have to rotate their body around a vertical axis in order to face in the sprinting direction. In the forward step technique, the vertical axis passes through the athlete's pivoting foot, while in the false step technique the axis passes through the body, closer to the COM. The mass moment of inertia is smallest when the rotational axis passes through the COM. The small mass moment of inertia in the false step technique compared with the forward step technique may provide an advantage for the false step technique.

In the sideward sprint start trials, one participant used only the forward step technique, but the others used both techniques across their three trials. In our previous study comparing sprint starts in a rightward direction, no significant difference was found in the time to perform a 5-m sprint between the false step and forward step techniques [15]. These results suggest that the two techniques provide similar sprint performance effects. In a backward or forward sprint start from the parallel stance, the athlete must move their COM anterior or step back to move the COP posterior in order to generate the needed COP-COM distance relative to the sprinting direction. In a sideward sprint start, however, the COP-COM distance can be generated by lifting the foot in the sprinting direction. A false step in a sideward sprint start increases the COP-COM distance and may increase acceleration, but the benefit may be eliminated by the time taken for the redundant step [15]. This is one possible reason for the conflicting results between the backward and sideward sprint starts.

There are two major limitations in this study as compared to observing a competitive soccer game. First, although sprinting was self-initiated in the present study, athletes typically start sprinting based on visual cues, such as moves of the opponents or ball. Second, the starting posture was static in this study. During a competitive soccer game, sprinting is started from another movement, such as jogging or walking, because athletes continue to move and never stop. Further study using a more closely simulated game condition is needed to reveal the step technique habitually used by athletes in a competitive game, and to further compare the superiority of these two step techniques. The findings in the present study regarding the step technique used in a self-initiated sprint start could be used as an important reference for further studies to assess the effects of types of visual cues or movements that are followed by sprinting. In addition, the results in the present study may be specific to high-level male soccer players. Therefore, different step technique preferences may be observed in athletes in other sports and possibly in female athletes, including female soccer players, because the muscle characteristics of females differ from those of males. Further studies focusing on female athletes and athletes in other sports are warranted to clarify sports-related adaptations or sex differences in the choice of step technique.

In the present study, we clarify the step techniques habitually used to start sprinting in a backward and a sideward direction. The results demonstrate that high-level male soccer players habitually use the false step technique in a backward sprint start and use both techniques with similar frequencies in a sideward sprint start. It was suggested that the choice tendency is different depending on the sprint start direction. The mechanical characteristics of these step techniques, as discussed as a reason of choice in the present study, might be evinced by our planned research on mechanical analyses of these movements.

## Declarations

### Author contribution statement

Takahiko Sato: Conceived and designed the experiments; Analyzed and interpreted the data; Contributed reagents, materials, analysis tools or data; Wrote the paper.

Yusuke Fukuhara: Conceived and designed the experiments; Performed the experiments; Analyzed and interpreted the data.

Tadao Isaka: Conceived and designed the experiments; Analyzed and interpreted the data; Contributed reagents, materials, analysis tools or data.

### Funding statement

This research did not receive any specific grant from funding agencies in the public, commercial, or not-for-profit sectors.

### Data availability statement

The data that has been used is confidential.

### Declaration of interests statement

The authors declare no conflict of interest.

### Additional information

No additional information is available for this paper.

## References

[1] Andrzejewski M., Chmura J., Pluta B., Konarski J.M. (2015). Sprinting activities and distance covered by top level Europa league soccer players. Int. J. Sports Sci. Coach..

[2] McLellan C.P., Coad S., Marsh D., Lieschke M. (2013). Performance analysis of super 15 rugby match-play using portable micro-technology. J. Athl. Enhanc..

[3] Rivilla-García J., Calvo L.C., Jiménez-Rubio S., Paredes-Hernández V., Muñoz A., van den Tillaar R., Navandar A. (2019). Characteristics of very high intensity runs of soccer players in relation to their playing position and playing half in the 2013-14 Spanish La Liga season. J. Hum. Kinet..

[4] Keogh J.W.L., Weber C.L., Dalton C.T. (2003). Evaluation of anthropometric, physical, and skill-related tests for talent identification in female field hockey. Can. J. Appl. Physiol..

[5] Reilly T., Williams A.M., Nevill A., Franks A. (2000). A multidisciplinary approach to talent identification in soccer. J. Sports Sci..

[6] Brown T., Vescovi J. (2004). Is stepping back really counterproductive?. Strength Condit. J..

[7] Cronin J., Green J., Levin G., Brughelli M., Frost D. (2007). Effect of starting stance on initial sprint performance. J. Strength Condit Res..

[8] Cusick J.L., Lund R.J., Ficklin T.K. (2014). A comparison of three different start techniques on sprint speed in collegiate linebackers. J. Strength Condit Res..

[9] Frost D., Cronin J.B., Levin G. (2008). Stepping backward can improve sprint performance over short distances. J. Strength Condit Res..

[10] Frost D., Cronin J. (2011). Stepping back to improve sprint performance: a kinetic analysis of the first step forwards. J. Strength Condit Res..

[11] Johnson T., Brown L., Coburn J., Judelson D., Khamoui A., Tran T., Uribe B. (2010). Effect of four different starting stances on sprint time in collegiate volleyball players. J. Strength Condit Res..

[12] Knudsen N.S., Andersen T.B. (2017). The effect of first-step techniques from the staggered stance in American football. Sports Med. Int. Open..

[13] LeDune J., Nesser T., Finch A., Zakrajsek R. (2012). Biomechanical analysis of two standing sprint start techniques. J. Strength Condit Res..

[14] Kraan G.A., van Veen J., Snijders C.J., Storm J. (2001). Starting from standing; why step backwards?. J. Biomech..

[15] Sato T., Fukuhara Y., Fujimoto M., Isaka T. (2018). Forward and false step techniques used for sprint start in a sideways direction: which is superior?. ISBS Proceedings Archive.

